# Duplication of *OsHAP* family genes and their association with heading date in rice

**DOI:** 10.1093/jxb/erv566

**Published:** 2016-01-21

**Authors:** Qiuping Li, Wenhao Yan, Huaxia Chen, Cong Tan, Zhongmin Han, Wen Yao, Guangwei Li, Mengqi Yuan, Yongzhong Xing

**Affiliations:** ^1^National Key Laboratory of Crop Genetic Improvement, Huazhong Agricultural University,Wuhan 430070, China; ^2^Hubei Collaborative Innovation Center for Grain Industry, China

**Keywords:** Association analysis, expression profiling, gene duplication, heading date, nucleotide diversity, *OsHAP*.

## Abstract

Heterotrimeric Heme Activator Protein (HAP) family genes are involved in the regulation of flowering in plants. It is not clear how many *HAP* genes regulate heading date in rice. In this study, we identified 35 *HAP* genes, including seven newly identified genes, and performed gene duplication and candidate gene-based association analyses. Analyses showed that segmental duplication and tandem duplication are the main mechanisms of *HAP* gene duplication. Expression profiling and functional identification indicated that duplication probably diversifies the functions of *HAP* genes. A nucleotide diversity analysis revealed that 13 *HAP* genes underwent selection. A candidate gene-based association analysis detected four *HAP* genes related to heading date. An investigation of transgenic plants or mutants of 23 *HAP* genes confirmed that overexpression of at least four genes delayed heading date under long-day conditions, including the previously cloned *Ghd8*/*OsHAP3H*. Our results indicate that the large number of *HAP* genes in rice was mainly produced by gene duplication, and a few *HAP* genes function to regulate heading date. Selection of *HAP* genes is probably caused by their diverse functions rather than regulation of heading.

## Highlight

Gene duplication has led to the existence of a large *HAP* gene family. In this study, three HAP genes were identified that regulate flowering in rice in addition to the previously reported *Ghd8/OsHAP3H*.

## Introduction

The heterotrimeric Heme Activator Protein (HAP) complex is also known as the CCAAT box factor (CBF) or nuclear factor Y (NF-Y) (Mantovani, 1999). The HAP complex consists of three subunits: HAP2 (NF-YA; CBF-B), HAP3 (NF-YB; CBF-A), and HAP5 (NF-YC; CBF-C). This complex binds to CCAAT sequences in a promoter to control the expression of target genes (Gusmaroli *et al.*, 2001; Kusnetsov *et al.*, 1999). In animals and yeast, there is a single gene for each HAP subunit, while in plants, there are gene families encoding each subunit. For example, in rice there are 10 genes (*OsHAP2A*–*OsHAP2J*) encoding the OsHAP2 unit, 11 genes (*OsHAP3A*–*OsHAP3K*) encoding the OsHAP3 unit, and seven genes (*OsHAP5A*–*OsHAP5G*) encoding the OsHAP5 unit (Thirumurugan *et al.*, 2008).

Recent studies have revealed the function of members of the HAP family in multiple plant developmental processes. *LEAFY COTYLEDON 1* (*LEC1*) and *LEC1-LIKE* (*LIL*), which share similar sequences, regulate embryogenesis in Arabidopsis (Kwong *et al.*, 2003; Lotan *et al.*, 1998). Additionally, *NF-YA3* and *NF-YA8* are functionally redundant genes that are required in the early embryogenesis of Arabidopsis (Fornari *et al.*, 2013). The expression of *NF-YA5* is strongly induced by drought stress and ABA treatments in Arabidopsis (Li *et al.*, 2008). Overexpression of the *AtNF-YB7* transcription factor confers drought tolerance and improves water-use efficiency in Arabidopsis (Han *et al.*, 2013). Overexpression of *AtNF-YB1* and *ZmNF-YB2* has been shown to increase seed grain yield and improve performance under drought conditions (Nelson *et al.*, 2007). The CCT (CONSTANS, CO-like, and TOC1) domain of CO in Arabidopsis and the functional domain conserved within HAP2 share important residues, and CO might replace AtHAP2 to form a trimetric CO/AtHAP3/AtHAP5 complex in the HAP complex. Overexpression of *AtHAP2* or *AtHAP3* delays flowering in Arabidopsis (Wenkel *et al.*, 2006). *AtHAP3b* is required to regulate flowering time in Arabidopsis under osmotic stress conditions (Chen *et al.*, 2007).

In rice, several CCT domain family genes have been confirmed to regulate heading date, such as major flowering regulators of *Ghd7* (*Grain number, plant height and heading date 7*), *Ghd7.1*, and *Hd1* (Xue *et al.*, 2008; Yan *et al.*, 2013; Yano *et al.*, 2000). *Hd1* regulates the day-length oscillations of *Hd3a* mRNA and promotes flowering under short-day conditions while inhibiting flowering under long-day conditions (Hayama *et al.*, 2003). Both *Ghd7* and *Ghd7.1* delay flowering by suppressing expression of *Ehd1* under long-day conditions (Xue *et al.*, 2008; Yan *et al.*, 2013). The functions of the *HAP* genes have been also characterized in rice. *OsHAP3A*, *OsHAP3B*, and *OsHAP3C* control chloroplast biogenesis (Miyoshi *et al.*, 2003). *OsLEC1*/*OsHAP3E* participates in the determination of meristem identity in both vegetative and reproductive development (Zhang and Xue, 2013). *OsHAP2E* confers resistance to pathogens, salinity, and drought, and increases photosynthesis and the tiller number (Alam *et al.*, 2015). *OsNF-YB1*, a rice endosperm-specific gene, encodes a cell cycle regulator and plays a role in maintaining endosperm cell proliferation (Sun *et al.*, 2014). The evidence from these various studies shows that the *OsHAP* family genes might have important and diverse functions in plant development and abiotic stress tolerance. However, only one *HAP* gene, *Ghd8*/*OsHAP3H,* acts upstream of *Ehd1, Hd3a*, and *RFT1* to regulate heading in rice (Yan *et al.*, 2011b). It is not clear whether other *HAP* family genes are functional in rice heading.

Heading date has been well studied in rice during the past two decades. Hundreds of quantitative trait loci (QTLs) for heading date have been collected on the Gramene website (http://www.gramene.org/, last accessed: 14 September 2013). Association mapping is an efficient approach to establish the relationship between molecular markers and traits in a given population based on linkage disequilibrium (LD) (Flint-Garcia *et al.*, 2003). Genome-wide association studies have gained prominence in recent years, especially in species with a rapid LD decay. For example, maize has a LD decay of 1–5kb, which can map QTL to a single gene (Remington *et al.*, 2001). The extent of LD variation is greater in *japonica* (150–167kb) than *indica* (75–123kb) rice, and LD patterns vary among genomic regions (Huang *et al.*, 2010; Mather *et al.*, 2007). Association mapping can be adversely affected by many factors, including population structure and small sample size, which may increase the detection of false-positive associations (Yan *et al.*, 2011a). Huang *et al.* (2010, 2012) made a genome-wide association mapping study for heading date in rice, but only a small number of cloned flowering genes were identified, even when using different populations and analysis methods. However, candidate gene association studies remain a key approach to gene mapping due to their high efficiency (Ehrenreich *et al.*, 2009; Zhang *et al.*, 2015).

The aim of the present study was to determine how many *HAP* family genes potentially have a function in the regulation of heading date. A total of 529 rice accessions were used to test the association between the *HAP* family genes and heading date. Then, we overexpressed or suppressed 18 *HAP* family genes in *japonica* rice Zhonghua 11 and Hejiang 19 (containing *Ghd8*/*OsHAP3H*) and tested the mutants of nine *HAP* genes under long-day and short-day conditions. Additionally, to elucidate how the *HAP* family genes have evolved, we analyzed the nucleotide diversity and the fixation index (Fst) of population differentiation. Our results showed that 13 *HAP* genes underwent selection and that at least four *HAP* genes regulate heading date in rice, including the previously cloned gene *Ghd8*/*OsHAP3H*.

## Materials and methods

### Plant materials, field experiments, and heading date

A total of 529 rice (*Oryza sativa*) cultivars, comprising a Chinese core collection consisting of 203 varieties except C126 and a world core collection of 330 accessions except W190, W196, and W232, and 107 common wild rice (*Oryza rufipogon*) accessions were sown in the field of an experimental farm of Huazhong Agricultural University, Wuhan, on 18 May 2011 and 19 May 2012. Basic information for the 529 cultivars is available on the RiceVarMap website (http://ricevarmap.ncpgr.cn/, last accessed: 10 August 2014) and information for the 107 wild rice accessions was previously reported by Zhang *et al.* (2015). Seven plants of each genotype were planted in a one-row plot at distances of 16.5cm within a row and 26.4cm between rows. Field management was in accordance with normal agricultural practices. Five plants in the middle of a row were used to score heading date; and the average date across the five plants was used as the heading date of the genotype for association mapping (see below). The heading dates for the 529 cultivars in 2011 and 2012 are presented in Supplementary Table S1 at *JXB* online.

### Search for *HAP* family genes

To obtain sequencing data for all of the *HAP* genes in rice, BLASTP searches were performed in the predicted protein databases of the rice genome TIGR (http://rice.plantbiology.msu.edu/, last accessed: 10 August 2014) and NCBI (http://www.ncbi.nlm.nih.gov/, last accessed: 10 August 2014) with partial HAP proteins as queries (Thirumurugan *et al.*, 2008). If a protein sequence satisfied *E*<10^−10^, it was selected as a candidate HAP protein. The SMART (http://smart.embl-heidelberg.de/, last accessed: 10 August 2014) and Pfam (http://pfam.sanger.ac.uk/, last accessed: 10 August 2014) databases were then used to predict the domain of all the candidate proteins. The deduced nucleotide and protein sequences of the new *HAP* genes in rice were downloaded from the TIGR database. In addition to the 28 *HAP* family genes that were reported by Thirumurugan *et al.* (2008), seven further *HAP* genes were identified; these were named sequentially according to their genome positions (Supplementary Table S2). To construct the phylogenetic tree of the HAP proteins, multiple protein sequences were executed using MEGA version 4.0 software to generate a maximum parsimony tree with bootstrapping analysis (Tamura *et al.*, 2007).

### Analysis of the expansion patterns of HAP genes

The method developed by Maher *et al.* (2006) was used to identify segmental duplications between *HAP* genes. First, we constructed a phylogenetic tree and identified all of the paralogous *HAP* genes at the terminal nodes. Next, 10 protein-coding genes that were upstream and downstream of each pair of paralogs were obtained from Gramene (Ware *et al.*, 2002). Finally, the genes flanking one *HAP* gene were matched to the genes flanking the other *HAP* gene in the same pair. If these sequences resided within a region of conserved protein-coding genes, the paralogous *HAP* gene pair was regarded as the result of a duplication event. Tandem duplications were arbitrarily defined as ones that occur within a sequence distance of 50kb (Riechmann *et al.*, 2000). The homology among the duplicated genes was calculated using MEGA version 4.0 software.

### Nucleotide diversity and evolution analysis

The whole genomic DNA sequences of the 529 cultivar accessions and 107 *O. rufipogon* accessions were genotyped with approximate two-fold coverage genome sequencing using a bar-coded multiplex sequencing approach on an Illumina Genome Analyzer II (Chen *et al.*, 2014). We extracted genomic sequences, defined as the DNA sequence of the gene from the transcription initiation site to the transcription stop site, of all *HAP* family genes from the TIGR database (http://rice.plantbiology.msu.edu/, last accessed: 10 August 2014). A sequence approximately 2kb in length, upstream of the genomic sequence, was extracted and considered to encompass the promoter region.

Two parameters of nucleotide diversity (the expected heterozygosity per nucleotide site, π, and theta per site from Eta, θ) were calculated. Tajima’s *D* statistic (Tajima, 1989) was used to search for evidence of selection. We calculated the ratio of genetic diversity in wild rice to that in cultivated rice (π_w_/π_c_) across genes to screen for selection signals. Fst, a standardized measure of the genetic variance between populations, was analyzed. All sequence analyses were conducted using DnaSP version 5.00 software (Librado and Rozas, 2009).

### Expression profile analysis

The expression profile data of some duplicated *OsHAP* genes in *indica* rice Zhenshan 97 were extracted from the CREP database (http://crep.ncpgr.cn/, last accessed: 4 January 2015), including 25 RNA samples from several tissues at different developmental stages.

### Candidate gene-based association mapping

For association analysis, the genome sequences of the 529 *O. sativa* accessions were downloaded from RiceVarMap. Using the population structure and relative kinship as covariates, single nucleotide polymorphism (SNP)–trait associations were analyzed with TASSEL software, using a mixed linear model. Analyses were conducted with population structure estimates, using the Q-matrix obtained from RiceVarMap. The parameter of the number of ancient clusters, K, was set from two to seven to obtain different inferences. The Bonferroni-adjusted significance threshold (*P*<0.05/n) was used to identify significant associations (n polymorphic sites) (Xu *et al.*, 2011).

### Transformation of HAP family genes

We amplified cDNAs of 18 *OsHAP* family genes (including *Ghd8*/*OsHAP3H*) from Nipponbare seedlings with gene-specific primers (Supplementary Table S3) using the high-fidelity LA Taq polymerase (Takara, Otsu, Japan). The cDNA of *OsHAP* genes was cloned into the binary vector pCAMBIA1301S-XBZ with the 35S promoter. Meanwhile, we used a 150–250bp sequence of part of the 3′ section of the target gene with the application of the double-stranded RNAi vector ds1301 to reduce expression of the gene. Additionally, the fragments of *OsHAP3H*, *OsHAP2A*, *OsHAP2B*, *OsHAP2E*, *OsHAP2K*, *OsHAP5A* and *OsHAP5B* for RNAi were identified by sequencing with gene-specific primers (Supplementary Table S3) and inserted into the binary vector ds1301, which inhibited the expression of RNA. All the recombinant constructs were delivered to wild type Zhonghua 11 (ZH11) or Hejiang 19 (HJ19) callus according to the rice genetic transformation method (Lin and Zhang 2005). The rice plants were grown in a paddy field under natural environmental conditions in Wuhan. Three mutants, *Oshap2c*, *Oshap2j*, and *Oshap2e*, were obtained from the Rice Mutant Database (http://rmd.ncpgr.cn/, last accessed: 14 October 2013), and six mutants, *Oshap2h*, *Osha2d*, *Oshap3l*, *Oshap5b*, *Oshap5d*, and *Oshap5h*, were obtained from the Rice T-DNA Insertion Sequence Database (http://an6.postech.ac.kr/pfg/index.php, last accessed: 14 October 2013).

### Identification of positive transgenic plants

We extracted the total DNA from fresh leaves by using the CTAB method (Kidwell and Osborn, 1992). To identify positive transgenic plants, we first amplified the *GUS* fragment from the transgenic plants using the primers GUSF+GUSR. Positive transgenic plants were identified by the presence of a bright band 1.5kb in size on gel electrophoresis. Then, we randomly selected positive and negative transgenic plants checked by *GUS* amplification to detect the level of expression of the target genes. These two methods in combination enabled us to identify positive and negative plants.

### RNA extraction and quantitative real-time reverse transcription PCR

Total RNA was extracted from leaves using an RNA extraction kit (TRIzol reagent, Invitrogen) following the manufacturer’s instructions. First-strand cDNA was reverse transcribed from DNase I-treated RNA with oligo(dT) as the primer. The expression of *OsHAP5A, OsHAP5B*, *OsHAP3D* and *OsHAP3E* was measured by quantitative real-time reverse transcription PCR (qRT-PCR). Approximately 3mg total RNA was reverse transcribed using M-MLV reverse transcriptase (Invitrogen) in a volume of 180 µl to obtain cDNA. The *Ubiquitin* gene was used as an internal control for the qRT-PCR. The primers used for qRT-PCR are listed in Supplementary Table S3. qRT-PCR was run in a total volume of 15 µl containing 3.6 µl of the reverse-transcribed product obtained as described above, 0.25mM gene-specific primers and 7.8 µl FastStart Universal SYBR Green Master (Rox) superMIX (Roche, Mannheim, Germany) on an Applied Biosystems ViiA 7 RT-PCR system, according to the manufacturer’s instructions. The relative quantification method was used to measure the quantity of qRT-PCR product. The qRT-PCR was performed using the following program: 95 °C for10min, then 40 cycles of 95 °C for 5s and 60 °C for 34s.

## Results

### Identification of *HAP* family genes

**Fig. 1.**
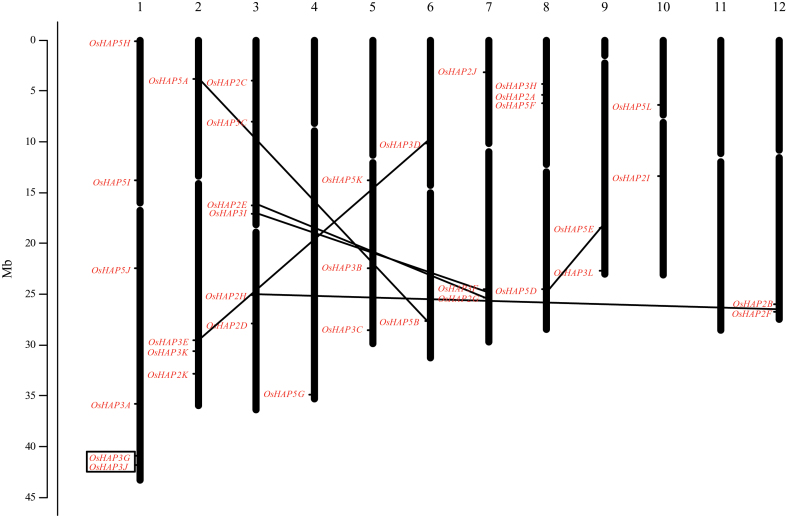
Genetic linkage map indicating the positions of *HAP* family genes. The *HAP* genes are indicated on the chromosomes in red. The segmental duplication events corresponding to *HAP* genes are indicated by black lines between the chromosomes. The black-outlined box indicates tandem duplication events.

In addition to the 28 genes that were previously reported by Thirumurugan *et al.* (2008) in rice, we identified one additional *HAP2* gene, one additional *HAP3* gene, and five additional *HAP5* genes (Supplementary Table S2). The newly identified genes were termed *OsHAP2K*, *OsHAP3L*, and *OsHAP5H*–*OsHAP5L*. The 35 *HAP* family genes included 11 *HAP2*, 12 *HAP3*, and 12 *HAP5* genes. *HAP* genes were distributed on every chromosome except chromosome 11 (Fig.1). Chromosomes 1 and 3 each harbored the largest number (six) of H*A*P genes; there were four *HAP* genes on chromosomes 2 and 8; chromosomes 5 and 7 contained three *HAP* genes; chromosomes 6, 9, 10, and 12 each contained two *HAP* genes; and chromosome 4 carried a single *HAP* gene. A phylogenetic tree was constructed with a maximum parsimony method using protein sequences that were deduced from Nipponbare (Supplementary Fig. S1A). The newly identified *HAP* genes were grouped into their corresponding subfamilies, indicating that they are real *HAP* family genes.

### Structure of *HAP* genes and proteins

All the members of the *HAP* family identified in rice each had a complete HAP domain (Supplementary Fig. S1B). All the other OsHAP proteins contained only a CBFB_NFYA or CBFD_NFYB domain, except *OsHAP5F*, which included the pollen_allerg_1 and transmembrane domains. Analysis of the gene structure showed that 17 (48.6%) of the 35 *OsHAP* genes were intronless. *OsHAP2* genes each had four to six exons, the *OsHAP3* genes had one to three exons, and the *OsHAP5* genes each had one exon, except *OsHAP5F*, which had six exons (Supplementary Fig. S1C).

### Duplication of *HAP* family genes

A total of 10 pairs of rice paralogous genes were identified on the terminal node of the phylogenetic tree (Supplementary Fig. S1A). No highly conserved genes were observed between the flanking regions of the paralogous pairs of *OsHAP2B*/*2D*, *OsHAP3B*/*3C*, and *OsHAP5I*/*5J*, indicating that these pairs of paralogous genes were formed through random translocation and insertion events. However, the conserved genes were detected between the flanking regions of other paralogous pairs of *OsHAP2E*/*2G*, *OsHAP2F*/*2H*, *OsHAP3D*/*3E*, *OsHAP3F*/*3I*, *OsHAP5A*/*5B*, and *OsHAP5D*/*5E* (Table 1). Therefore, these paralogous genes might have arisen from segmental duplications. *OsHAP3G* and *OsHAP3J,* with 49.6% homology (Fig.1, Supplementary Table S4), were located in a tandem repeat on chromosome 1, indicating that tandem duplication events contributed to producing these two genes. There are therefore seven pairs of paralogs in duplicate (Fig.1, Supplementary Table S4). We compared the expression patterns of three pairs of genes, *OsHAP2E/2G* and *OsHAP3E/3D*, which represented segmental duplication patterns, and *OsHAP3G/3J*, which represented a tandem duplication pattern. The segmentally duplicated pair of *OsHAP3D* and *OsHAP3E* showed similar expression patterns in 25 tested stages/tissues (Supplementary Fig. S2A). In contrast, the expression patterns of the segmentally duplicated pair of *OsHAP2E* and *OsHAP2G* and the tandem duplication pair of *OsHAP3G* and *OsHAP3J* were different in most of these tissues (Supplementary Fig. S2B, C). qRT-PCR analysis for eight tissues at different developmental stages showed consistent results (Supplementary Fig. S3).

**Table 1.** Conserved protein-coding genes in their flanking regions of segmentally duplicated *HAP* genes

**Duplicated gene 1**	**Conserved protein-coding flanking gene 1**	**Duplicated gene 2**	**Conserved protein-coding flanking gene 2**	**Annotation for flanking genes**
*OsHAP2E*	*Os03g29830*	*OsHAP2G*	*Os07g41660*	Expressed protein
*OsHAP2F*	*Os12g42420*	*OsHAP2H*	*Os03g44580*	DNA binding protein
*OsHAP3D*	*Os06g17410*	*OsHAP3E*	*Os02g49440*	dof zinc finger domain
*OsHAP3F*	*Os07g41600*	*OsHAP3I*	*Os03g29920*	proline-rich protein
*OsHAP5A*	*Os02g07430*	*OsHAP5B*	*Os06g45650*	MADS-box with MIKCc type-box
*OsHAP5D*	*Os08g38810*	*OsHAP5E*	*Os09g30320*	BURP domain

### Nucleotide diversity of *HAP* genes and evolution analysis

The genomic DNA length of the 35 *OsHAP* genes ranged from 377–9357bp. We analyzed the DNA polymorphisms of the *HAP* family genes within 107 wild rice accessions and 529 cultivars (Supplementary Tables S5–7). There were 998, 496, and 856 SNPs within the HAP2, HAP3, and HAP5 subfamilies, respectively, across the entire sequence (containing the promoter and genomic sequences) among the 529 cultivar accessions (Table 2). The average nucleotide diversity of whole *HAP* family genes in both *O. sativa* and wild rice (π=3.3×10^−3^) was higher than that of the whole genome in *O. sativa* (π=2.4×10^−3^) (Huang *et al.*, 2012). In the promoter region, the nucleotide diversity was not significantly different among the *HAP2*, *HAP3*, and *HAP5* subfamilies. However, compared with the *HAP2* and *HAP5* subfamilies, the *HAP3* subfamily genes, on average, demonstrated low nucleotide diversity in both *O. sativa* and *O. rufipogon* (π= 1.4×10^−3^ and 1.6×10^−3^, respectively) in the genomic sequence. The average nucleotide diversity (π=4.2×10^−3^) was thus apparently higher in the promoter regions of the 35 *HAP* family genes than in the genomic sequence (Table 2). No difference was observed between *O. sativa* and *O. rufipogon* for each subfamily.

**Table 2.** Nucleotide diversity of *HAP* family genes in cultivar and wild rice

***HAP* family**		**Cultivars**			**Wild rice**		
		**SNPs**	**π (×10** ^−**3**^)	**θ (×10** ^−**3**^)	**SNPs**	**π (×10** ^−**3**^)	**θ (×10** ^−**3**^)
Promoter	*HAP2*	398	4.7	2.2	470	4.6	3.2
	*HAP3*	370	3.9	2.1	533	4.2	3.4
	*HAP5*	376	4.0	2.3	525	4.7	3.3
	Whole	1144	4.2	2.2	1528	4.5	3.3
Genomic sequence	*HAP2*	600	2.6	1.3	703	2.5	1.9
	*HAP3*	126	1.4	0.8	184	1.6	1.5
	*HAP5*	480	3.1	1.9	505	3.2	2.7
	Whole	1206	2.4	1.3	1392	2.4	2.0
Entire sequence	*HAP2*	998	3.7	1.8	1173	3.6	2.6
	*HAP3*	496	2.7	1.5	717	2.9	2.5
	*HAP5*	856	3.6	2.1	1030	4.0	3.0
	Whole	2350	3.3	1.8	2920	3.5	2.7

Estimates of nucleotide diversity were calculated based on average pairwise diversity (π) and the number of segregating sites (θ). The promoter region has a length of 2kb upstream of the gene.

The pairwise nucleotide diversity in the genomic sequence ranged from 0–11 SNPs per kb (Supplementary Tables S5–7). *OsHAP2A* and *OsHAP5J* had the highest diversity, with approximately 8–11 SNPs per kb in both *O. sativa* and wild rice. *OsHAP2C*, *OsHAP2J, OsHAP3B*, *OsHAP3E*, *OsHAP5D*, and *OsHAP5E* had the lowest diversity, with less than 1 SNP per kb in the genomic sequence. In the promoter region, the pairwise nucleotide diversity ranged from 1–14 SNPs per kb, higher than in the genomic sequence. *OsHAP2E* and *OsHAP5J* had the highest diversity in the promoter region, with approximately 9–14 SNPs per kb in both *O. sativa* and wild rice. In cultivated rice, the Tajima’s *D* values in the genomic sequence of *OsHAP2A*, *OsHAP2B*, *OsHAP2E*, *OsHAP3C*, *OsHAP3L*, and *OsHAP5J* reached a significantly positive level; in contrast, no gene in wild rice reached a significantly positive level. The π_*w*_/π_*c*_ ratio in the promoter region and in the genomic sequences of *OsHAP2J* and *OsHAP5B* was greater than 4 (Supplementary Tables S5–7), indicating that these genes underwent selection during domestication and genetic improvement. For most of the 35 *OsHAP* family genes, the population-differentiation statistic (Fst) between the populations of *indica* (295 varieties) and *japonica* (156 varieties) was greater than 0.25 (Supplementary Table S8), indicating a very strong population differentiation. Only *OsHAP2J*, *OsHAP3D, OsHAP3F*, *OsHAP5B, OsHAP5E*, and *OsHAP5F* had a lower Fst between the *indica* and *japonica* groups.

### Association between heading date and *HAP* family genes

Four genes, including three *OsHAP3* and one *OsHAP5* subfamily genes, were significantly associated with heading date at *P*<10^−3^ under long-day conditions in both 2011 and 2012 (Table 3). No genes in the *OsHAP2* subfamily were associated with heading date. The associated gene contained the previously cloned flowering QTL *Ghd8*/*OsHAP3H* (Yan *et al.*, 2011b). The associated SNP site of *OsHAP5A* fell into an intron region. *OsHAP3E* has the same identity as *OsLEC1*, a key regulator of meristem identity determination in both vegetative and reproductive development (Kwong *et al.*, 2003; Lotan *et al.*, 1998). *OsHAP3G* exhibited the highest significant association in 2011, at *P*=3.2×10^−11^. The associated SNP sites of two, one, and one genes were located in untranslated region, intron, and exon regions, respectively.

**Table 3.** *HAP* family genes associated with heading date

**Gene**	**2012**		**2011**	
	**Association site**	***P***	**Association site**	***P***
*OsHAP3G*	3′UTR	5.6×10^–3^	3′UTR	3.2×10^–11^
*OsHAP3E*	Exon	2.1×10^–3^	Exon	2.4×10^–3^
*OsHAP3H* (*Ghd8*)	5′UTR	2.5×10^–5^	5′UTR	1.3×10^–3^
*OsHAP5A*	Intron	2.1E×10^–3^	Intron	7.9×10^–3^

*P* values of the association signals were calculated from the mixed linear model.

3′UTR, 3′untranslated region; 5′UTR, 5′ untranslated region.

### Phenotype of the mutants of nine *HAP* family genes

To rapidly confirm the function of the *HAP* family genes in relation to heading date, we searched the database in Rice GE (http://signal.salk.edu/cgi-bin/RiceGE/, last accessed: 14 October 2013) and found 11 distinct mutants that targeted 11 *HAP* family genes. However, only nine mutants of *OsHAP* genes were germinated and planted, due to poor seed viability. We performed two sets of PCR to genotype each mutation, one using a pair of gene-specific primers (F and R) and the other using a gene-specific primer (F) and a corresponding T-DNA border primer (TRB2 or PGAR2) (Supplementary Fig. S4, Supplementary Table S3). However, none of the mutants had a change in heading date compared with that of wild-type plants under either long-day or short-day conditions (Supplementary Table S9).

### Flowering function identification of 18 *HAP* family genes by transformation

**Fig. 2.**
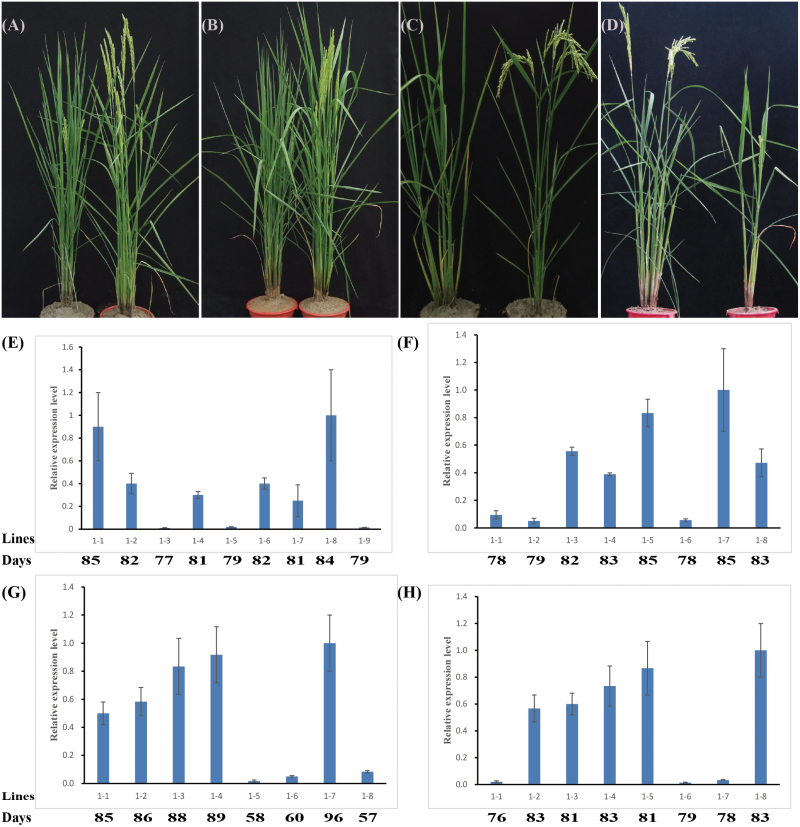
Performance in heading date of transgenic plants of four *HAP* genes and real-time PCR analyses. (A) Overexpression plants of *OsHAP5A* (left) and wild-type Zhonghua 11 (right) in the T_1_ generation. (B) Overexpression plants of *OsHAP5B* (left) and wild-type Zhonghua 11 (right) in the T_1_ generation. (C) Overexpression plants of *OsHAP3E* (left) and wild-type Hejiang 19 (right) in the T_0_ generation. (D) Overexpression plants of *OsHAP3D* (right) and wild-type Zhonghua 11 (left) in the T_0_ generation. (E–G) qRT-PCR analyses of (E) *OsHAP5A* and (F) *OsHAP5B* in the T_1_ generation, and of (G) *OsHAP3E* and (H) *OsHAP3D* in the T_0_ generation. The numbers beneath each graph show the number of days to heading for the plants. The relative expression levels were normalized with that of rice *Ubiquitin*. The highest level was set at 1.

Among the four associated genes, *OsHAP3H*/*Ghd8* has previously been confirmed to regulate heading date (Yan *et al.*, 2011). Thus, we tested the function of the additional three genes by overexpression. In addition, to determine whether the non-associated *HAP* family genes affect heading date, we randomly overexpressed 14 genes—*OsHAP2C*, *OsHAP2G*, *OsHAP2I*, *OsHAP5B*, *OsHAP5E*, *OsHAP2B*, *OsHAP2E*, *OsHAP2H*, *OsHAP2K*, *OsHAP3D*, *OsHAP3B*, *OsHAP3C*, *OsHAP5H*, and *OsHAP5K*—in *japonica* rice ZH11 or HJ19 (Fig. 2). The positive *OsHAP5A* (Fig. 2A) and *OsHAP5B* (Fig. 2B) plants had significant differences in heading date compared with negative (wild-type) plants in the T_1_ generation under long-day conditions (Table 4). Moreover, significant differences in heading date were observed between negative and positive *OsHAP3E* (Fig. 2C) and *OsHAP3D* (Fig. 2D) plants in the T_0_ generation under long-day conditions (Table 4). qRT-PCR analyses indicated that in the overexpression lines of *OsHAP5A* (Fig. 2E), *OsHAP5B* (Fig. 2F), *OsHAP3E* (Fig. 2G), and *OsHAP3D* (Fig. 2H), the mRNA levels of these four genes were indeed higher than in wild-type plants. Co-segregation analysis in the T_1_ generation showed that overexpression of *OsHAP5A* (Chi-square=0.04, *P*=0.8) and *OsHAP5B* (Chi-square=0.4, *P*=0.5) resulted in delayed heading (Table 4). *OsHAP3E* and *OsHAP3D* overexpression lines were sterile, but all positive plants in the T_0_ generation were always heading later than wild-type and negative plants (Table 4). *OsHAP5A* and *OsHAP3D* delayed heading by 3–4 days under long-day conditions. *OsHAP5B* delayed heading by approximately 7 days. *OsHAP3E* greatly delayed the heading date of HJ19, by 30 days. 

**Table 4.** Heading date of transgenic plants for the functional *HAP* genes under long-day conditions

**Gene**	**Overexpression lines**	**Positive plants**		**Negative plants**		**P1**	**Chi-square**	**P2**
		**No**	**HD (d**)	**No**	**HD (d**)			
*OsHAP3D*	OX (ZH11)-T_0_	5	82.2±1.1	3	77.7±1.5	1.2×10^–3^		
*OsHAP3E*	OX (HJ19)-T_0_	5	88.8±4.3	3	58.3±1.5	2.65×10^–5^		
*OsHAP5A*	OX (ZH11)-T_1_	23	81.8±4.3	7	78.3±1.0	5.3×10^–6^	0.04	0.8
*OsHAP5B*	OX (ZH11)-T_1_	24	83.6±2.8	6	77.8±1.6	4.0×10^–11^	0.4	0.5

OX (HJ19) and OX (ZH11) represent the overexpressed plants of the Hejiang 19 and Zhonghua 11 genotype, respectively. No, number of plants investigated; HD, heading date. P1 values were obtained by *t* tests for significant difference in heading date between the positive and negative plants. P2 values were obtained by chi-squared test for segregation of 3:1. T_0_ plants came from different resistant callus. T_1_ plants came from one T_0_ plant.

In addition, we constructed *OsHAP2A*, *OsHAP2B*, *OsHAP2E*, *OsHAP2K*, *OsHAP3H*, *OsHAP5A*, and *OsHAP5B* for double-stranded RNA lines in the ZH11 background. However, the RNAi plants of *OsHAP2A*, *OsHAP2B*, *OsHAP2E*, *OsHAP2K*, *OsHAP3H*, *OsHAP5A*, and *OsHAP5B* did not show any change in heading date from the wild type. 

Except for *OsHAP3E*, *OsHAP3D*, *OsHAP5A* and *OsHAP5B*, the other 13 overexpressed genes did not change heading date (Supplementary Table S10). In addition, these nine mutants, *Oshap2c*, *Oshap2d*, *Oshap2e*, *Oshap2h*, *Oshap2j*, *Oshap3l*, *Oshap5b*, *Oshap5d*, and *Oshap5h*, did not exhibit any change in heading date.

Some heading genes in rice were previously reported to function under short-day conditions (Yano *et al.*, 2000; Yan *et al*. 2011b). Thus, we investigated the heading dates of all transgenic plants, mutants, and their controls covering 20 *HAP* genes under short-day conditions (winter Hainan). None of the tested *HAP* genes functioned under short-day conditions except *Ghd8*/*OsHAP3H* (Supplementary Table S10).

## Discussion

### At least four *HAP* genes including *Ghd8*/*OsHAP3H* control heading date in rice

In this study, we identified four *HAP* family genes that were associated with heading date, based on a diverse germplasm collection. We then tested the function of 18 *HAP* genes on heading date by overexpressing or silencing cDNAs from Nipponbare seedlings and nine mutants that were targeted at *HAP* genes. The overexpression of *OsHAP5A* and *OsHAP5B* genes delayed heading date under long-day conditions in the T_1_ generation. *OsHAP3D* and *OsHAP3E* delayed heading date under long-day conditions in the T_0_ generation; for these genes, data was not obtained in the T_1_ generation because of the transgenic were sterile plants. All five of the positive *OsHAP3E* plants headed 30 days later than the negative plants (Table 4), indicating with certainty that *OsHAP3E* regulates heading date. For *OsHAP3D*, although the difference in heading between positive and negative plants in T_0_ was only four day*s,* all five of the positive plants consistently headed later than the negative plants, suggesting that *OsHAP3D* might regulate heading date. These genes belonged to the subfamilies encoding HAP5 and HAP3 subunits, in addition to our previously cloned *Ghd8*/*OsHAP3H*. No HAP2 subfamily genes regulated heading date. It should be noted that only Nipponbare alleles were used to test the function of *HAP* genes in this study. We cannot be sure that all the Nipponbare alleles are functional, although no premature termination mutation was observed in the Nipponbare alleles (Supplementary Table S11). Alternatively, overexpressing the alleles from other genotypes for these *HAP* genes, which were presumed non-functional in regulating heading by overexpressing Nipponbare alleles, may identify more *HAP* genes that regulate heading. In addition, we tested only 23 of the 35 identified *HAP* genes, and the remaining 13 genes need to be tested in future. From these findings we can conclude that at least four HAP genes, including *Ghd8*/*OsHAP3H*, regulate heading date in rice.

It is surprising that none of the nine tested HAP2 subfamily genes regulated heading date. In Arabidopsis, the HAP proteins have been suggested to work in a complex composed of HAP2/HAP3/HAP5 (Ben-Naim *et al.*, 2006). Even though the additional two untested HAP2 genes, *OsHAP2D* and *OsHAP2F*, do not regulate heading date, this result is also understandable because the CCT domain of CO can replace HAP2 and interact with HAP3/HAP5 to regulate flowering in Arabidopsis (Wenkel *et al.*, 2006). There are dozens of CCT family genes that are involved in the flowering pathway in rice (Zhang *et al.*, 2015), providing flexibility to form a heterotrimeric complex.

### Type I and type II errors in association mapping were identified by transformation

Four genes were associated with heading date, but one of them was non-functional in regulating heading date. Additionally, no association was detected between *OsHAP5B* or *OsHAP3D* and heading date. However, *OsHAP5B*- and *OsHAP3D*-overexpressing plants showed a significantly delayed heading date. These results indicated that both type I and type II errors occurred when performing the association analysis. In statistics, a type I error is the incorrect rejection of a true null hypothesis. In general, a type I error is frequently caused by low threshold values; before running an association analysis, the exclusion of rare causal mutations (i.e. SNPs) due to low frequency will also lead to a type I error. Similar to *OsHAP3H* (*Ghd8*), the causal mutation is a SNP that is located +322bp from the initiation codon ATG, resulting in a premature stop in protein translation (Yan *et al.*, 2011b). Because this SNP is detected in only a few accessions, it was not included in the association analysis. Therefore, the SNP was not identified as being associated with heading date. A type II error is the failure to reject a false null hypothesis. In general, a type II error is frequently caused by high threshold values, but a close linkage between the tested *HAP* family genes and a QTL/gene influencing heading date is a possible reason for the type II error here because LD decay is slow in rice, extending to an approximately 200kb region (Huang *et al.*, 2012). For example, the association between *OsHAP3G* and heading date is probably due to linkage to the heading date gene *OsHY2* (Saito *et al.*, 2010), because *OsHAP3G* itself was verified to be non-functional in regulating heading. In cases of type I and type II errors, general linear models and linear mixed models are recommended for association analysis, besides specifying a reasonable threshold value for claiming an association.

### Overexpression enhanced the power of functional identification of genes in a large family

Overexpression and knockout/knockdown mutations have been confirmed to be efficient ways to identify gene function. In general, mutants are primarily recommended for testing gene function. However, the members of a large gene family frequently have a similar function in plants, and loss of function of one gene can be rescued by other members of the gene family (Hughes and Liberles, 2007). Consequently, gene function frequently cannot be identified for a single gene mutant in a large gene family. For example, in this study, for *OsHAP5A* and *OsHAP5B*, the overexpression lines significantly delayed heading date, but the RNAi lines showed no change under long-day conditions. Similar to *OsHAP5A* and *OsHAP5B*, *Ghd8*/*OsHAP3H* was confirmed to suppress flowering in rice, increasing plant height and yield potential simultaneously (Yan *et al.*, 2011b), but *Ghd8*-RNAi lines did not alter heading date, plant height or yield (Supplementary Table S10). It is obvious that some redundant genes exist in the rice HAP gene family. This was the case in a previous study identifying the function of CCT family genes (Zhang *et al.*, 2015). Although RNAi plants cannot adequately unveil the function of HAP family genes, they can be used to identify redundant genes by crossing them to produce double or triple mutants. Considering the redundancy among a large number of genes within a family, overexpression easily validates gene function and is largely encouraged to use for testing the gene function of a large gene family.

### Duplication diversified the function of *HAP* family genes

Three mechanisms contribute to the expansion of gene families—tandem duplication, large-scale block duplication (segmental duplication), and transposition events, such as retro-transposition and replicative transposition (Kong *et al.*, 2007). The duplication of protein-coding genes can result in loss of function (pseudogenes), maintenance of the original function (redundant function), or acquisition of a new function (neofunctionalization) (Hughes and Liberles, 2007). There are 18 pairs of duplicated segments that together cover 65.7% of the rice genome (Yu *et al.*, 2005). In this study, among 10 pairs of paralogous *HAP* family genes (Supplementary Fig. S1A), one pair (*OsHAP3G* and *OsHAP3J*) was identified as having formed under tandem duplication. Six pairs of *HAP* genes were segmentally duplicated. Among these genes, four pairs (*OsHAP3F*/*3I*, *OsHAP2E*/*2G*, *OsHAP5D*/*5E*, and *OsHAP5A*/*5B*) were located on the corresponding duplication blocks in the rice genome; the two pairs of *OsHAP3D*/*3E* and *OsHAP2F*/*2H* were not. Both of these latter pairs probably arose from duplication events and lost their counterparts over a long period of evolution, because the rice genome underwent ancient polyploidization through two rounds of polyploidy events followed by massive gene losses and numerous chromosome rearrangements (Guyot and Keller, 2004).

Part of the short arm of chromosome 2 harboring *OsHAP5A* and part of the long arm of chromosome 6 harboring *OsHAP5B* (Fig. 1) include pairs of duplicated segments of the rice genome (Yu *et al.*, 2005). After this duplication, *OsHAP5A* and *OsHAP5B* maintained the same function in regulating heading date, which can be explained by the mechanisms of concerted evolution and purifying selection (Li, 2006; Nei *et al.*, 2000). Similarly, the duplicated genes *OsHAP3D* and *OsHAP3E* play critical roles in embryogenesis (Kwong *et al.*, 2003; Lotan *et al.*, 1998) and flowering regulation. Similar expression profiles of *OsHAP3D* and *OsHAP3E* were also observed (Supplementary Fig. S2A). These findings indicate that some segmentally duplicated genes in the *OsHAP* family maintained conserved functions during evolution. The expression patterns of the *OsHAP2E*/*2G* pair were different in most of the tissues that were tested (Supplementary Fig. S2B), indicating that one member of the duplicate might have gained a new function. The tandem-duplicated genes *OsHAP3G* and *OsHAP3J* demonstrated a different expression pattern in most tissues, indicating a functional differentiation. Therefore, the *HAP* genes have evolved to have diverse functions such as drought stress tolerance (Han *et al.*, 2013) and pathogen resistance (Alam *et al.*, 2015), and only a few *HAP* genes regulate heading in rice.

### Diverse function contributed to the selection of *HAP* family genes

The selective signatures from domestication have been applied most commonly to data from genes (Doebley *et al.*, 2006). In this study, we found that HAP genes had a higher Fst between *indica* and *japonica* subspecies, indicating that the genes in both populations are significantly differentiated. A positive Tajima’s *D* signifies that population stratification or balancing selection occurred in this locus during the evolution and breeding of rice. A negative Tajima’s *D* signifies an excess of low-frequency polymorphisms relative to expectations, indicating a selective sweep or purifying selection. Significant positive Tajima’s *D* values indicated that at least 13 genes, including *OsHAP2A*, *OsHAP2B*, *OsHAP3L*, and *OsHAP5J,* were subject to balancing selection or population stratification during the natural evolutionary process. Moreover, *OsHAP2B* was located in a selective sweep region (Huang *et al.*, 2012). However, we did not observe any function of *OsHAP2B* in relation to heading date. These results indicate that a strong selection pressure was imposed on the region of *OsHAP2B* during domestication of rice. The selection of *OsHAP2B* was probably the result of genetic ‘hitch-hiking’.

When gene mutation affects the fitness and survival of an organism, selection naturally occurs during evolution. In particular, when a mutation leads to a trait that is desirable by humans, this mutation is artificially selected after the species has been domesticated (Sang, 2009). Therefore, it is expected that some *HAP* family genes that underwent selection would be responsible for important biological and agronomic traits. In this study, only four genes regulated heading date, which is an important factor when determining ecological adaptation. In addition, *OsHAP2E* confers resistance to pathogens, salt, and drought, and increases photosynthesis and tiller number (Alam *et al.*, 2015). It is likely that some *HAP* genes may have a potential function in abiotic stress tolerance and have consequently been selected during domestication and genetic improvement. It would be worthwhile to test the stress tolerance of *HAP* genes using these transgenic lines in future.

## Supplementary data

Supplementary data are available at *JXB* online.

Table S1. Heading date data for 529 cultivars in 2011 and 2012.

Table S2. List of 35 HAP family genes identified in rice.

Table S3. Primers used for amplifying gene fragments and transcriptional expression analysis.

Table S4. Homology among duplicated *HAP* genes.

Table S5. DNA polymorphic sites of *HAP2* genes.

Table S6. DNA polymorphic sites of *HAP3* genes.

Table S7. DNA polymorphic sites of *HAP5* genes.

Table S8. F statistics of *HAP* family genes between *japonica* and *indica* cultivars.

Table S9. Heading dates of mutants targeted nine HAP family genes.

Table S10. Functional identification on heading date of 23 *HAP* family genes by transformation or mutant in long-day condition (Wuhan) and short-day condition (Hainan).

Table S11. Variation in the position of the stop codon in Nipponbare allele in 529 rice cultivars.

Fig. S1. Phylogenetic tree for rice *HAP* family and protein structure of OsHAP proteins.

Fig. S2. Comparison of expression profiles between duplicated *OsHAP* genes.

Fig. S3. qRT-PCR analysis of the expression of six *OsHAP* genes.

Fig. S4. PCR tests for mutants *Oshap2j* (A) and *Oshap5d* (B) as an example.

Supplementary Data
