# Supplementary material for: Duplication of OsHAP family genes and their association with heading date in rice
Source: J Exp Bot. 2016 Jan 21;67(6):1759–68. doi: 10.1093/jxb/erv566 (PMC4783360; doi:10.1093/jxb/erv566)
Supplement: Supplementary Data [file supp_67_6_1759__index.html]

Duplication of OsHAP family genes and their association with heading date in rice — Supplementary Data 

# Duplication of *OsHAP* family genes and their association with heading date in rice

## Supplementary Data

Data files

- supplementary\_tables\_S1.xlsx - Supplementary Data
- supplementary\_tables\_S2\_S11\_figures\_S1\_S4.pdf - Supplementary Data
